# Supplementary material for: Transcriptome-wide association studies: recent advances in methods, applications and available databases
Source: Commun Biol. 2023 Sep 1;6:899. doi: 10.1038/s42003-023-05279-y (PMC10474133; doi:10.1038/s42003-023-05279-y)
Supplement: Supplementary file 2 — Supplementary Figure 1 [file 42003_2023_5279_MOESM2_ESM.pdf]

Transcriptome-wide association studies: recent advances in methods, applications and available databases

Jialin Mai[1,2,3,#], Mingming Lu[1,2,3,#], Qianwen Gao[1,2,3], Jingyao Zeng[1,2,*], Jingfa Xiao[1,2,3,*]

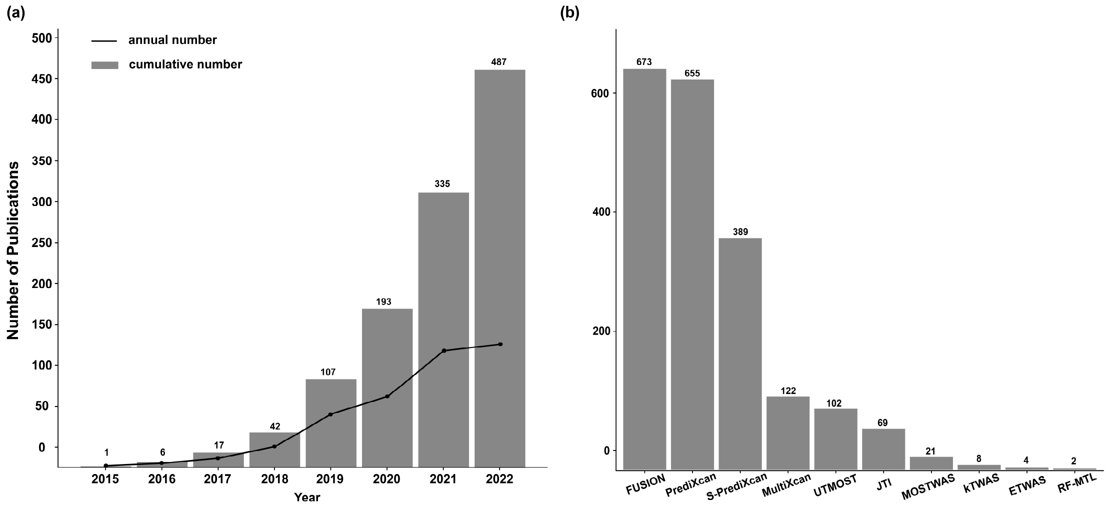

Supplementary Figure 1. Statistics of TWAS publications. a Statistics of TWAS-related research in recent years. The line represents the increased number each year, and the bar represents the sum number by then. b Distribution of publications according to TWAS methods (until December 2022).
